# Dual Roles of Heterotrophic Ammonia-Oxidizing Bacteria in Enhancing Compensatory Growth upon Post-Drought in Maize

**DOI:** 10.3390/microorganisms12122383

**Published:** 2024-11-21

**Authors:** Hao Yu, Xiao-Ling Wang, Run-Hong Sun, Lin Qi, Peng Song, Tong-Chao Wang

**Affiliations:** 1 College of Agronomy, Henan University of Science and Technology, Luoyang 471023, China; 17838406839@163.com (H.Y.); qilin19850515@163.com (L.Q.); songpeng19800852@163.com (P.S.); 2 Henan Key Laboratory for Control of Crop Diseases and Insect Pests, IPM Key Laboratory in Southern Part of North China for Ministry of Agriculture, Institute of Plant Protection Research, Henan Academy of Agricultural Sciences, Zhengzhou 450099, China; sunrunhongwxl@163.com; 3 College of Agriculture, Henan Agricultural University, Zhengzhou 450046, China; wtcwrn@126.com

**Keywords:** heterotrophic ammonia-oxidizing bacteria, maize, rhizosphere soil nitrification, cytokinin

## Abstract

This study investigates the mechanisms driving maize compensatory growth upon post-drought, to reveal how the root’s original cytokinins are regulated by the two-fold roles of heterotrophic bacteria with ammonia-oxidizing (HAOB) capabilities. The HAOB’ dual roles encompass influencing root cytokinin synthesis and transport through nitrification and a direct pathway. Experiment 1 involved introducing the application of varying amounts of NO_3_^−^ to the roots to examine how nitrification affects cytokinin roots-to-leaves transport. Results demonstrate that the 30–40 mmol·L^−1^ NO_3_^−^ concentration had ideal effects on enhancing post-drought growth in maize by facilitating cytokinin synthesis and transport. In experiment 2, an HAOB strain, S2_8_1, was utilized and NO_3_^−^ was supplemented alongside HAOB inoculation to assess the joint impacts of nitrification and the direct pathway on the production and transportation of cytokinins. Results demonstrate that the HAOB strain S2_8_1 increases nitrification rates in rhizosphere soil, thereby promoting the transport of cytokinins from roots to leaves. In addition, the HAOB strain promotes root cytokinin transport to leaves autonomously, showcasing its direct pathway. Inoculation with the HAOB strain increased leaf cytokinin content and improved water use efficiency compared to the addition of NO_3_^−^; however, the combination of NO_3_^−^ and HAOB strains resulted in a synergistic effect and further improvement. These findings elucidate how HAOB can enhance maize compensatory growth through its dual roles, presenting promising applications in agriculture.

## 1. Introduction

Water shortage presents a critical challenge to global agricultural production, jeopardizing crop yields and food security [1,2]. Enhancing water use efficiency in agriculture is key to mitigating this issue. Drought stress often hampers crop growth [3,4,5]. However, timely water restoration can trigger compensatory growth that accelerates their growth and offsets the losses caused by drought stress [6,7,8,9]. Consequently, water-saving techniques like supplemental irrigation, deficit irrigation, and controlled deficit irrigation, based on the compensatory growth theory, are widely adopted to improve water efficiency in agriculture [10,11]. Understanding the mechanisms behind compensatory growth after drought rewatering is thus pivotal in addressing the global water scarcity crisis.

Post-drought compensatory growth in crops is characterized by rapid growth triggered by rewatering [12,13,14,15]. Research by Wang et al. (2020) indicates that rewatering induces maize root systems to produce cytokinins, which are then transported to the leaves through the xylem sap to regulate the compensatory growth [16]. Crucially, nitrate nitrogen (NO_3_^−^) serves as a pivotal trigger for the synthesis of cytokinins in roots. Upon detecting NO_3_^−^ in the soil, plant roots initiate the cytokinin synthesis process [17,18]. The NO_3_^−^ primarily originates from soil nitrification, a series of biochemical processes involving soil microorganisms. Among these microorganisms, ammonia-oxidizing bacteria are crucial in determining the nitrification rate [19,20]. Wang et al. (2021, 2022) observed that an ammonia-oxidizing bacteria strain exhibiting heterotrophic nutritional traits significantly enhances nitrification rated in rhizosphere soil, thereby promoting the soil NO_3_^−^ release, accelerating the cytokinin roots-to-leaves transport, and consequently fostering maize compensatory growth upon post-drought rewatering [21,22]. Therefore, heterotrophic ammonia-oxidizing bacteria (HAOB) in soils are very important for crops’ post-drought compensatory growth.

In addition to soil nitrate nitrogen, certain soil bacteria can directly induce cytokinin production in plant roots. These bacteria, present in the rhizosphere, can modulate cytokinin levels and distribution within the host plant [23,24]. Furthermore, the same bacteria may possess multiple functions, such as influencing nutritional traits and secreting plant hormones. Consequently, other pathways of HAOB could contribute to cytokinin production in maize roots. Hence, we hypothesize that HAOB influences root cytokinin production and translocation to leaves via dual roles: nitrification and another pathway. Confirming this hypothesis would reveal a new mechanism by which soil microorganisms influence a crop’s rewatering compensatory growth following drought, potentially leading to the development of new technologies for water-saving and drought-resistant crop cultivation techniques.

To test this hypothesis, the HAOB strain S2_8_1, preserved at the Typical Culture Conservation Center of China, was utilized in this study, with maize chosen as the plant material. Maize, the third largest crop globally, is highly susceptible to drought stress and subsequent rewatering, making it an ideal candidate for observing the HAOB strain’s impact on promoting compensatory growth after drought. Excessive NO_3_^−^ was introduced to the roots to evaluate the HAOB strain’s maximum effect on root cytokinin delivery via rhizosphere nitrification. Co-application of excessive NO_3_^−^ and HAOB inoculation was employed to investigate whether they independently stimulate cytokinin synthesis in leaves.

## 2. Materials and Methods

### 2.1. Experimental Design

We opted for the HAOB strain S2_8_1, which is part of the genus *Ensifer* and is conserved at the China Center for Type Culture Collection in Wuhan under the accession CCTCC NO: M2021374, GenBank database number ON667919. The strain was isolated and screened from soil at the farm of Henan University of Science and Technology in Luoyang City, Henan Province, China. In this study, a liquid medium containing 0.5 g (NH_4_)_2_SO_4_, 0.75 g KH_2_PO_4_, 0.25 g NaH_2_PO_4_, 0.01 g MnSO_4_·4H_2_O, 0.03 g MgSO_4_·7H_2_O, and 5.0 g CaCO_3_ dissolved in 1 L of distilled water was used with the pH adjusted to 7.2 [22]. The pure colony was inoculated into the liquid medium and sealed and fermented for seven days under protection from light. The HAOB bacterial liquid was used in this experiment. The concentration of the bacterial solution was measured by UV spectrophotometer.

The maize (*Zea mays* L.) cultivar “Zhengdan 958” was selected for potting experiments because it is known for its drought resistance and because the maize grows so fast in the seedling stage that changes can be clearly observed.

This study conducted a potting experiment at Henan University of Science and Technology’s farm in Luoyang City, Henan Province, China, positioned at 34°32′ N, 112°16′ E, and 138 m above sea level. The region experiences an average yearly precipitation of 600 mm and temperature of 14.1 °C. On 15 May 2023, fifteen seeds of maize were planted in plastic pots. There were 400 such plastic pots, each measuring 20 cm in diameter, standing 25 cm high. Each pot held 5.75 kg of brown soil containing 23.6 g·kg^−1^ of organic carbon and 2.2 g·kg^−1^ of total nitrogen. The maize seedlings sprouted approximately 6 days after planting. Then, 6 days after sprouting, the seedlings were spaced out, leaving five robust healthy plants per pot.

The remaining five seedlings in each pot grew for 5 days. On the 11th day after emergence, seventy-two pots with uniform, healthy, and robust seedlings were chosen for experiment 1 (Exp-1), and sixty pots were selected for experiment 2 (Exp-2). Over the subsequent 10 days, consistent watering and lighting were provided to maximize the reduction of experimental variability. Between the 21st and 31st day after emergence, a drought period lasting 10 days was implemented. Then, between the 32nd and 42nd day after emergence, a rewatering period lasting 10 days was established.

#### 2.1.1. Experiment 1

In experiment 1, 72 pots were used, with each pot receiving around 30 mL of a 3,4-dimethylpyrazole phosphate (DMPP) solution (1.5 g·L^−1^) daily, commencing three days before the conclusion of the drought period. The rationale behind incorporating DMPP was derived from prior research [25,26], aiming to hinder soil nitrification, thereby reducing soil NO_3_^−^ availability and paving the way for subsequent effects of added NO_3_^−^.

These 72 pots were divided into 6 groups (treatments): DN (post-drought rewatering only), DN-1, DN-2, DN-3, DN-4, and DN-5 (post-drought rewatering with NO_3_^−^ at concentrations of 10, 20, 30, 40, and 50 mmol·L^−1^, respectively). Each group was further divided into 4 subgroups, with 3 pots per subgroup. During the rewatering period, the DN treatment received water only, while DN-1 to DN-5 received the corresponding NO_3_^−^ concentrations to ensure adequate water supply.

#### 2.1.2. Experiment 2

Sixty pots in experiment 2 were categorized into six groups, each corresponding to a specific treatment: (1) regular water supply (WA); (2) rewatering following drought (WB); (3) rewatering following drought with NO_3_^−^ solution of 35 mmol·L^−1^ (WN); (4) rewatering following drought with 100 mL of S2_8_1 bacterial solution (WJ); and (5) rewatering following drought with NO_3_^−^ solution of 35 mmol·L^−1^ and 100 mL of S2_8_1 bacterial solution (NJ). Each treatment was divided into four subgroups, with each subgroup containing three pots.

The WA treatment consistently received ample water supply during both the drought stress period and subsequent rewatering period. Alternatively, the other treatments experienced drought conditions during the drought period and received adequate water supply during the subsequent rewatering phase. The inclusion of liquid media, with or without the inclusion of AOB (216,090 cfu·mL^−1^), occurred at the onset of the rewatering phase. Throughout the rewatering phase, an additional solution containing NO_3_^−^ was provided to the WN and NJ treatments to ensure sufficient water supply. Water was supplied to the WA, WB, and WJ treatments to maintain this condition.

#### 2.1.3. Soil Moisture Regulation

According to previous research [27], soil moisture conditions (75 ± 5% and 45 ± 5% of field capacity) were employed to ensure adequate water provision and induce drought stress in the present study. A 10 days drought period was chosen based on the reliability demonstrated in preliminary studies, which confirmed its effectiveness in inhibiting maize seedling growth without causing significant harm [28]. To regulate soil moisture levels within the desired range, water was supplemented whenever the moisture content in the containers dropped below 45% or rose above 75% of field capacity. Soil water content was determined by using Formula (1):

(1) $$SWC=\frac{B_{t}-B_{d}-B_{e}-B_{p}}{B_{d}\times FWC}\times 100\%$$

Here, SWC stands for soil water content. B_t_, B_d_, B_e_, and B_p_ stand for the total pot weight at a specific time, the mass of dried soil, the mass of empty pots, and the estimated live plant mass, respectively. FWC represents the soil’s water-holding capacity. Bp was determined using extra pots during the initial phase of the experiment.

### 2.2. Measurements

#### 2.2.1. Biomass, Water Use Efficiency, and Soil Nitrification Rate

The soil adhered to the root system was washed away with water. The roots, stems, and leaves obtained were oven-dried at 65 °C for 72 h to obtain dry biomass. Aboveground biomass was calculated by summing the dry weights of the stems and leaves, while total biomass included the dry weights of the roots, stems, and leaves. Water use efficiency was indicated by the increase in total biomass per pot divided by water consumption during the drought stress and rewatering periods. Transpiration rates (T_r_) were determined by an LI-6800 Photosynthesis analyzer from 10:00 am to 13:00 pm.

To obtain rhizosphere soil samples, pots were cut open, and the soil was extracted together with the roots. The roots were then shaken repeatedly until minimal soil remained attached. The fine soil grains gathered at this stage constituted the rhizosphere soil samples used in this research.

The NO_3_^−^ and NH_4_^+^ contents in the soil were measured by the phenol disulfonic acid colorimetric method and the indophenol blue colorimetric method, respectively [29]. Soil samples, adjusted to 60% of field water-holding capacity, were incubated at 25 °C for a week to assess the daily soil nitrate increment during the incubation period, from which soil nitrification rates were calculated.

#### 2.2.2. Zeatin Riboside

Maize stems were severed from the roots, and the incision was covered using 1.0 g of cotton wadding to soak xylem sap over 12 h. Enclosed in plastic film, the cotton prevented water loss through evaporation. The increase in cotton weight due to the absorption of xylem sap was divided by the density of water (1 g·cm^−3^) to obtain its volume. The volume collected per time unit represented the transport rate from roots to leaves. Afterward, the cotton wadding underwent repeated squeezing to collect the sap that was used to determine ZR concentration (C_ZR_) using enzyme-linked immunosorbent assay (ELISA), as per the method described by Qin and Wang (2020) [30]. Importantly, the test kits for zeatin riboside were produced at the Shanghai Enzyme-linked Biotechnology Co., Ltd. (Shanghai, China).

The ZR content in the leaves was expressed as the amount of ZR per unit leaf mass. Likewise, C_zr_ was calculated as the amount of ZR per unit sap volume. The delivery of root ZR to leaves was assessed in two ways: in the dark (B_ZR_) and under light (L_ZR_), using the following two equations [31].

(2) $${B_{ZR}=C}_{zr}\times X_{r}$$

(3) $${L_{ZR}=C}_{zr}\times T_{r}\times M\times SLA$$

Here, X_r_ stands for the xylem sap delivery rate in the absence of light, T_r_ represents plant transpiration rate, M represents the leaf biomass, and SLA (Specific Leaf Area) is the ratio of leaf surface area to dry weight.

#### 2.2.3. Data Analysis

Statistical analysis was performed with SPSS 27. The values depicted in the generated graphs represent the means. Soil nitrification rate, biomass, hormone levels, and nitrogen indices in the experiment were assessed via one-factor ANOVA followed by Duncan’s multiple comparison test (*p* = 0.05).

## 3. Results

### 3.1. Exp-1

**Figure 1:**
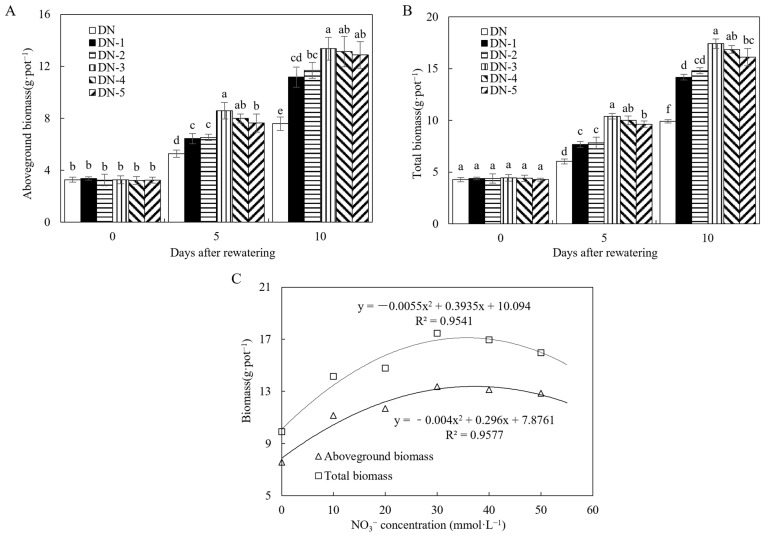
Biomass and regression analysis plot of experiment 1. (**A**) Aboveground biomass of experiment 1. (**B**) Total biomass of experiment 1. (**C**) Biomass regression analysis plot of experiment 1. DN: post-drought rewatering only; DN-1, DN-2, DN-3, DN-4, and DN-5: post-drought rewatering with NO_3_^−^ at concentrations of 10, 20, 30, 40, and 50 mmol·L^−1^, respectively. The numbers “0”, “5”, and “10” stand for the beginning, 5th, and 10th days of the 10 days rewatering period, respectively. The values are the mean ± standard error (*n* = 3). Different lowercase letters in the figure represent significant differences (*p* < 0.05).

In Figure 1, treatments DN-1 to DN-5 showed markedly increased aboveground and total biomasses in comparison with treatment DN at days 5 and 10 after rewatering. This demonstrates that NO_3_^−^ addition greatly enhanced maize growth following drought stress under rewatering conditions. Regression analysis further highlights that concentration between 30 and 40 mmol·L^−1^ exhibited the most significant enhancement on post-drought rewatering growth.

**Figure 2:**
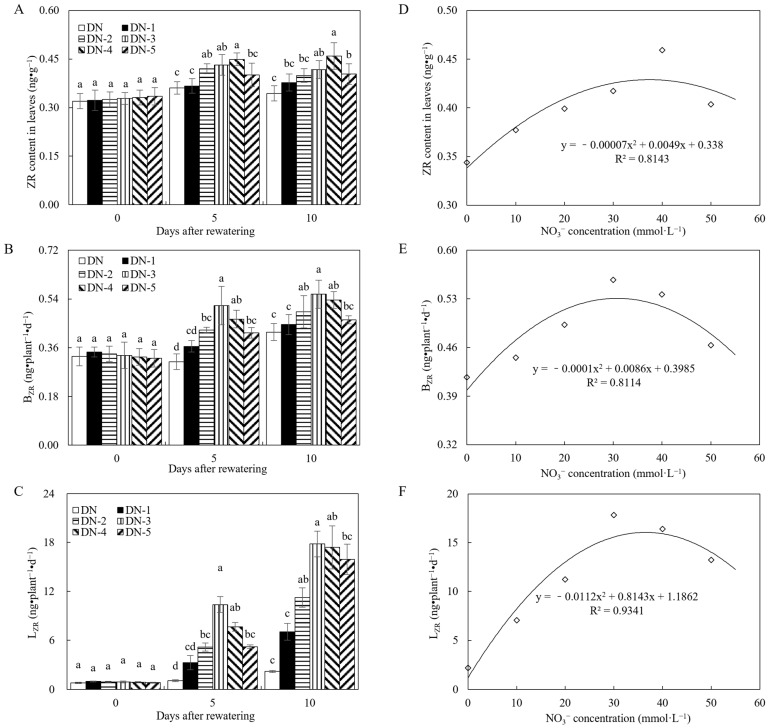
ZR, B_ZR,_ and L_ZR_ and their regression analysis plots of experiment 1. (**A**) ZR content in leaves of experiment 1. (**B**) B_ZR_ of experiment 1. (**C**) L_ZR_ of experiment 1. (**D**) ZR regression analysis plot of experiment 1. (**E**) B_ZR_ regression analysis plot of experiment 1. (**F**) L_ZR_ regression analysis plot of experiment 1. ZR represents cytokinin, BZR represents the transport rates of zeatin riboside from roots to leaves, which were calculated separately for darkness, LZR represents the transport rates of zeatin riboside from roots to leaves, which were calculated separately for darkness. DN: post-drought rewatering only; DN-1, DN-2, DN-3, DN-4, and DN-5: post-drought rewatering with NO_3_^−^ at concentrations of 10, 20, 30, 40, and 50 mmol·L^−1^, respectively. The numbers “0”, “5”, and “10” stand for the beginning, 5th, and 10th days of the 10 days rewatering period, respectively. The values are the mean ± standard error (*n* = 3). Different lowercase letters in the figure represent significant differences (*p* < 0.05).

In Figure 2, treatments DN-1 to DN-5 displayed significantly elevated ZR content in leaves relative to treatment DN at days 5 and 10 after rewatering. This phenomenon was also observed for B_ZR_ and L_ZR_. Given that ZR serves as a primary component of cytokinin, these findings suggest that NO_3_^−^ positively influenced the cytokinin levels in leaf tissues and facilitated their transport from roots to foliar tissue. Moreover, there appears to be an optimal NO_3_^−^ concentration range for this effect. Regression analysis revealed that NO_3_^−^ levels in the range of 30 to 40 mmol·L^−1^ had the greatest effect on these indices.

### 3.2. Exp-2

#### 3.2.1. Biomass

**Figure 3:**
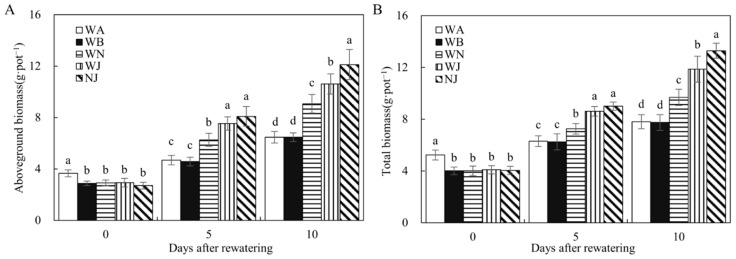
Biomass of experiment 2. (**A**) Aboveground biomass of experiment 2. (**B**) Total biomass of experiment 2. WA, WB, WN, WJ, and NJ represent regular water supply, post-drought rewatering, post-drought rewatering with addition of 35 mmol·L^−1^ of NO_3_^−^, post-drought rewatering with addition of 100 mL of S2_8_1 bacterial solution, and post-drought rewatering with addition of 35 mmol·L^−1^ of NO_3_^−^ and 100 mL of S2_8_1 bacterial solution, respectively. The numbers “0”, “5”, and “10” stand for the beginning, 5th, and 10th days of the 10 days rewatering period, respectively. The values are the mean ± standard error (*n* = 3). Different lowercase letters in the figure represent significant differences (*p* < 0.05).

Based on Figure 3, drought stress profoundly affects maize growth. By the conclusion of drought stress period, maize biomasses, both aboveground and total, were notably higher in treatment WA compared to other treatments. However, on the 5th and 10th days following rewatering, these biomasses of WA and WB were significantly lower than others, underscoring the pivotal role of NO_3_^−^ and the HAOB strain, whether used alone or together, in bolstering compensatory growth. The HAOB stain demonstrated a stronger effect than NO_3_^−^ alone, as evidenced by the total biomasses in treatment WJ surpassing that of treatment WN on days 5 and 10 post-rehydration. By day 10 after rehydration, treatment NJ exhibited significantly higher biomasses of both aboveground and total compared to WN and WJ treatments, indicating that the combined inoculation of NO_3_^−^ and HAOB stain substantially promoted compensatory growth.

**Figure 4:**
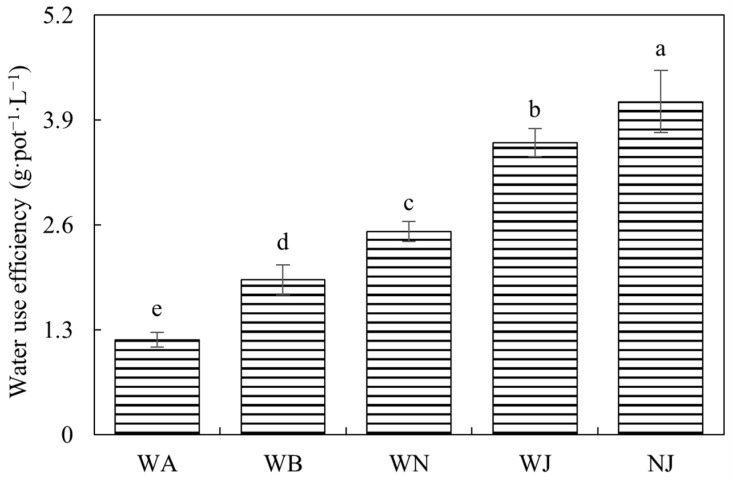
Water use efficiency in experiment 2. WA, WB, WN, WJ, and NJ represent regular water supply, post-drought rewatering, post-drought rewatering with addition of 35 mmol·L^−1^ of NO_3_^−^, post-drought rewatering with addition of 100 mL of S2_8_1 bacterial solution, and post-drought rewatering with addition of 35 mmol·L^−1^ of NO_3_^−^ and 100 mL of S2_8_1 bacterial solution, respectively. The values are the mean ± standard error (*n* = 3). Different lowercase letters in the figure represent significant differences (*p* < 0.05).

Figure 4 demonstrates that the water use efficiency of the WJ and NJ treatments significantly exceeded all others, with NJ exhibiting notably higher values compared to WJ. Additionally, the water use efficiency of the WN treatment significantly exceeded that of WA and WB. These findings underscore the substantial enhancement in water use efficiency attributed to both NO_3_^−^ and S2_8_1, with S2_8_1 demonstrating a particularly pronounced effect.

#### 3.2.2. Rhizosphere Soil Nitrification

**Figure 5:**
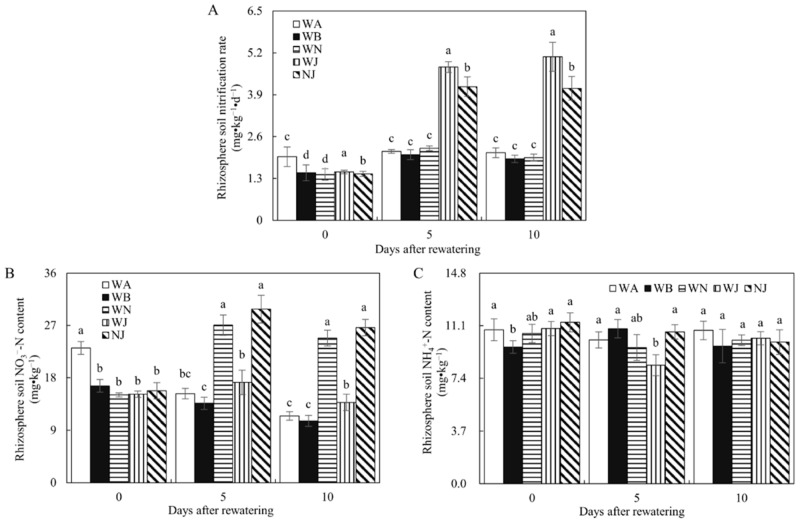
Rhizosphere soil nitrification rates, NO_3_^−^-N contents, and NH_4_^+^-N contents in soil of experiment 2. (**A**) Rhizosphere soil nitrification rates of experiment 2. (**B**) NO_3_^−^-N contents of experiment 2. (**C**) NH_4_^+^-N contents of experiment 2. WA, WB, WN, WJ, and NJ represent regular water supply, post-drought rewatering, post-drought rewatering with addition of 35 mmol·L^−1^ of NO_3_^−^, post-drought rewatering with addition of 100 mL of S2_8_1 bacterial solution, and post-drought rewatering with addition of 35 mmol·L^−1^ of NO_3_^−^ and 100 mL of S2_8_1 bacterial solution, respectively. The numbers “0”, “5”, and “10” stand for the beginning, 5th, and 10th days of the 10 days rewatering period, respectively. The values are the mean ± standard error (*n* = 3). Different lowercase letters in the figure represent significant differences (*p* < 0.05).

Figure 5 illustrates that the WJ and NJ treatments had significantly higher rhizosphere soil nitrification rates than the WA, WB, and WN treatments on days 5 and 10 after rewatering. No significant difference was observed among the WA, WB, and WN treatments. This suggests that the HAOB strain enhanced rhizosphere soil nitrification rates, while the addition of NO_3_^−^ did not have a similar effect. Furthermore, the rhizosphere soil nitrification rate increased significantly in WJ compared to NJ post-rewatering, implying that the addition of NO_3_^−^ could negatively affect this index under HAOB strain inoculation conditions. During rewatering, NO_3_^−^ addition significantly increased the rhizosphere soil NO_3_^−^ content in WN and NJ but had minimal impact on the rhizosphere soil NH_4_^+^ content.

#### 3.2.3. Zeatin Riboside

**Figure 6:**
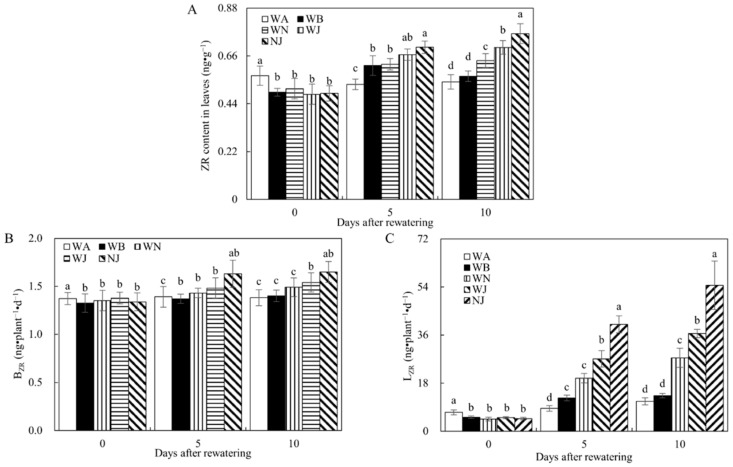
ZR, BZR, and LZR of experiment 2. (**A**) ZR content in leaves of experiment 2. (**B**) B_ZR_ of experiment 2. (**C**) L_ZR_ of experiment 2. ZR represents cytokinin, B_ZR_ represents the transport rates of zeatin riboside from roots to leaves, which were calculated separately for darkness, L_ZR_ represents the transport rates of zeatin riboside from roots to leaves, which were calculated separately for darkness. WA, WB, WN, WJ, and NJ represent regular water supply, post-drought rewatering, post-drought rewatering with addition of 35 mmol·L^−1^ of NO_3_^−^, post-drought rewatering with addition of 100 mL of S2_8_1 bacterial solution, and post-drought rewatering with addition of 35 mmol·L^−1^ of NO_3_^−^ and 100 mL of S2_8_1 bacterial solution, respectively. The numbers “0”, “5”, and “10” stand for the beginning, 5th, and 10th days of the 10 days rewatering period, respectively. The values are the mean ± standard error (*n* = 3). Different lowercase letters in the figure represent significant differences (*p* < 0.05).

As depicted in Figure 6, ZR content in leaves, B_ZR_, and L_ZR_ significantly decreased in the WA and WB treatments compared to WN, WJ, and NJ during the rewatering period. Thus, supplementing NO_3_^−^, inoculating with the HAOB strain, and their combined application increased cytokinin levels in leaves and facilitated roots-to-leaves cytokinin translocation. Notably, the impact of the HAOB strain was more pronounced than that of NO_3_^−^, as indicated by significantly higher ZR content in leaves in the WJ treatment compared to the WN treatment on day 10 post-rewatering; a similar trend was observed for B_ZR_ and L_ZR_. Moreover, the co-inoculation of the HAOB stain with NO_3_^−^ showed a more substantial facilitating impact compared to S2_8_1 alone. This was evident from the higher ZR content in leaves, B_ZR_, and L_ZR_ in the NJ treatment compared to the WJ treatment on day 10 following rewatering.

## 4. Discussion

### 4.1. Ideal Nitrate for Growth Enhancement

The NO_3_^−^ in the soil serves not only as the primary pathway for plants to acquire nitrogen nutrition but also plays another crucial physiological role—it induces the CTK synthesis in plant roots [32,33,34]. Specifically, when plant roots detect NO_3_^−^ in the soil, it initiates the synthesis process of CTK, which is subsequently transported to the leaves via the xylem sap, thereby regulating plant growth [35,36,37,38]. In the present study, NO_3_^−^ similarly performs this function.

In experiment 1, as shown in Figure 1, increasing the NO_3_^−^ concentration (0–30 mmol·L^−1^) resulted in higher values of B_ZR_ and L_ZR_, which then gradually plateaued within the range of 30–50 mmol·L^−1^. Through regression analysis, it was found that the optimal promotion effect of NO_3_^−^ occurs within its concentration range of 30–40 mmol·L^−1^. Elevated NO_3_^−^ concentration levels in the soil, such as 40–50 mmol·L^−1^, may lead to root congestion, potentially contributing to the observed peak in root cytokinin delivery to the foliage. Consequently, the specific concentration of 33.1 mmol·L^−1^ NO_3_^−^ resulted in the highest B_ZR_, while 36.4 mmol·L^−1^ NO_3_^−^ led to the highest L_ZR_, thereby amplifying the synthesis and translocation of root cytokinins to elevate cytokinin concentration in the foliage. This was further supported by the observation that the peak leaf ZR content of 35.0 mmol·L^−1^ also coincided with the concentration range of 30–40 mmol·L^−1^. Additionally, the peak total biomass of 35.8 mmol·L^−1^ also coincided with the concentration range of 30–40 mmol·L^−1^. Therefore, in the WN and NJ treatments in Exp-2, the selected NO_3_^−^ concentration (35.0 mmol·L^−1^) optimizes cytokinin transport from roots to leaves, enhancing subsequent growth-promoting effects during the rewatering period.

### 4.2. Dual Functions for Enhancing Growth

Under the rewatering phase of Exp-2, HAOB strain-inoculated treatments significantly enhanced the nitrification rate in rhizosphere soils, resulting in an average increase of 94% over untreated controls. This increased nitrification activity resulted in a greater release of soil NO_3_^−^, causing an average 1.61-fold rise in rhizosphere soil NO_3_^−^ content in the WJ treatment compared to WB. The surplus NO_3_^−^ availability facilitated the synthesis and continuous delivery of root cytokinins, indicated by higher levels of B_ZR_ and L_ZR_ in WJ over WB during rewatering. Consequently, the leaf cytokinin content showed an approximately 20% increase in the WJ treatment relative to the WB treatment during this period.

Building upon the findings of Exp-1, the selected NO_3_^−^ concentration (35 mmol·L^−1^) was shown in promoting cytokinin roots-to-leaves transport, in the WN treatment of Exp-2. However, a 35% higher L_ZR_ or 3% higher B_ZR_ was observed in WJ compared to WN during rewatering. The rise could not be solely ascribed to the impact of NO_3_^−^ resulting from HAOB strain-facilitated nitrification in the rhizosphere soils. Another process likely present, potentially related to the HAOB strain, directly enhances root cytokinin synthesis without relying on nitrification. Bacteria in the rhizosphere can influence the production of cytokinins in host plants [39,40]. Therefore, this study indicates that the HAOB strain may serve dual roles in enhancing cytokinin production in roots and facilitating their transport to the shoots. Consequently, these dual functions result in a 9% elevation in leaf cytokinin contents during rewatering in WJ compared to WN.

In the NJ treatment of Exp-2, besides NO_3_^−^, the HAOB strain promoted cytokinin transport from roots to leaves, resulting in BZR and LZR levels rising by more than 20% compared to the WN or WJ treatments during the regrowth period. As a result, NJ showed at least 17% higher leaf cytokinin contents than them. This emphasizes the somewhat separate role of the HAOB strain in directly increasing cytokinin transport, which is largely independent of its nitrification function. Wang (2021, 2022) [21,22] also found that the HAOB strain exerted a stronger influence on cytokinin roots-to-leaves transport, subsequently affecting leaf cytokinin levels in maize, compared to the addition of NO_3_^−^ to roots alone. This underscores the HAOB strain’s promotion of cytokinin upward transport through dual actions.

### 4.3. Implications for the Role of the HAOB Dual Pathway

Rewatering upon post-drought significantly enhanced nitrification in the maize rhizosphere, subsequently triggering the translocation of root-derived cytokinins to the shoot, which in turn promotes compensatory growth [16]. This phenomenon was also observed in our study. During the rewatering period, plants treated with WB demonstrated higher rhizosphere soil nitrification rates compared to those treated with WA. This led to the increased translocation of root-derived cytokinins to the leaves, fostering compensatory growth post-drought rewatering in the WB treatment and enhancing its water use efficiency by 62% compared to the WA treatment.

Similarly, the addition of NO_3_^−^ in WN, compared to MB, led to a greater cytokinin roots-to-leaves transport, resulting in higher cytokinin levels in leaves. This contributed to a 35% increase in total biomass and super-compensatory growth following drought rewatering, along with a 30% improvement in water use efficiency. Likewise, the heightened cytokinin levels in leaves in WJ, in comparison to MN, led to an over-90% increase in total biomass and a 40% enhancement in water use efficiency. These findings underscore the notable effect of the HAOB strain’s dual functions.

In actual crop water-saving production, fertilization is used to promote crop growth and development [41,42,43]. The impact of nitrogen fertilizer may encounter a bottleneck similar to NO_3_^−^ in this study. This is because most crop water-saving production occurs in dryland environments, where nitrogen fertilizer utilization primarily takes the form of NO_3_^−^, either through soil nitrification releasing NO_3_^−^ or through direct application of NO_3_^−^ [44,45]. Moreover, leveraging the dual mechanism of the HAOB strain could overcome the nitrogen fertilizer bottleneck, thereby making a greater contribution to crop water-saving production.

This dual mechanism not only promotes the water-saving growth of crops by providing plant hormones such as cytokinins but also supplies nitrogen nutrition through nitrification to support crop growth. This comprehensive enhancement of efficient water utilization in crops, from both the perspective of plant hormones and soil nitrogen supply, would be more advantageous. Rewatering growth is a rapid growth process that requires a substantial supply of nitrogen nutrition, with cytokinins playing a regulation role.

Nevertheless, the precise mechanism underlying the direct pathway of HAOB strains remains incompletely understood. We hypothesized that HAOB strains could directly interact with roots to enhance cytokinin synthesis. However, further studies are required to validate this hypothesis. If validated, it will substantially enhance our comprehension of the impacts of HAOB on crop growth and, consequently, its potential application in water-saving agriculture.

To deepen our understanding of these dual actions, it is essential to identify the alternative pathway besides nitrification. Clarifying the relationship between these two pathways is crucial. This will expand the theoretical implications of post-drought rewatering compensatory growth and, consequently, provide new directions for advancing agricultural water-saving technologies.

## 5. Conclusions

This study’s findings underscore the pivotal role of the HAOB strain in facilitating cytokinin roots-to-leaves transport, promoting compensatory growth through two distinct pathways: nitrification and another direct pathway. The HAOB strain enhances nitrification rates in rhizosphere soil, resulting in increased NO_3_^−^ release and enhanced cytokinin transport. Moreover, the HAOB strain exhibits a direct function, autonomously augmenting cytokinin transport independently of nitrate mediated by nitrification. Together, these mechanisms substantially elevate leaf cytokinin content, providing significant support for compensatory growth.

## Data Availability

These sequence data have been submitted to the GenBank databases under accession number ON667919, accessed on 1 June 2022. Addresses are as follow: https://ncbi.nlm.nih.gov/genbank.
